# Continuous glucose monitoring as a close to real life alternative to meal studies – a pilot study with a functional drink containing amino acids and chromium

**DOI:** 10.3389/fmedt.2022.931837

**Published:** 2022-08-18

**Authors:** Azat Samigullin, Per M. Humpert, Elin Östman

**Affiliations:** ^1^starScience GmbH, Heidelberg, Germany; ^2^Stoffwechselzentrum Rhein Pfalz, Mannheim, Germany; ^3^Department of Internal Medicine, Endocrinology and Diabetes, Heidelberg University Hospital, Heidelberg, Germany; ^4^Aventure AB, Lund, Sweden

**Keywords:** continuous glucose monitoring (CGM), postprandial glycemia, amino acids (AAs), meal study, glycemic index and glycemic load, dietary intervention glycemic control, dietary intervention randomized controlled trial, functional food (FF)

## Abstract

This pilot study aimed to evaluate a continuous glucose monitoring (CGM) based approach to study the effects of a functional drink containing specific amino acids and chromium picolinate (FD) and a combination of FD with a juice (FDJ) on postprandial glucose in a close to real life setting. The predefined primary endpoint for this study was the 120-min incremental area under the glucose curve (iAUC_0−120*min*_) after meals. It was estimated that using CGM and repeated meals in 6 participants could be sufficient to match the power of the previous study in regards to the quantity of meals. Participants followed a pre-specified meal schedule over 9 days and consumed the drinks three times daily with main meals. Differences between drinks were analyzed by analysis of covariances (ANCOVA) with subject number and activity as random factors and nutrient composition as covariates. In 156 meals available for analysis, a significant 34% reduction of glucose iAUC_0−120*min*_ was shown for FDJ (*p* < 0.001). FD did not show a significant effect on its own, but a significant reduction of 17.6% (*p* = 0.007) was shown in pooled data for FD and FDJ. While the differences between the two functional drinks used were not the primary focus of this study, it was sufficiently powered to detect previously described effects in 60 participants in a cross-over design under laboratory settings. The design presented defines a novel and cost-effective approach using CGM devices and app-based lifestyle tracking for studying nutritional effects on glucose at home in a close to real-life setting.

## Introduction

High glucose levels measured in a standardized oral glucose tolerance test are established, independent risk factors for type 2 diabetes (T2D), cardiovascular events and increased mortality, even below the diabetic and prediabetic thresholds (1–8). Pharmacological interventions targeting postprandial glycemia have been shown to be efficacious in preventing T2D, cardiovascular events and reducing mortality (9–11). Furthermore, health benefits of a general reduction of postprandial glucose and low glycemic index (GI) diets have been shown in association studies (12–17) and are backed up by experimental data looking at possible underlying mechanisms (18–21). Incremental area under the curve (iAUC_0−120*min*_) is an established measure for assessing the glycemic response in meal studies and for evaluating the GI of foods (22, 23).

In a previous double-blinded study it was shown that FD, flavored water containing the free amino acids leucine, isoleucine, valine, lysine and threonine, as well as chromium picolinate, was able to reduce the postprandial response (iAUC) to a carbohydrate-rich meal by 24% (24). These results were obtained in healthy, overweight individuals in a meal experiment under controlled laboratory settings (24). Being the gold standard for the assessment of postprandial glycemic response to different foods, meal studies require trained staff, are costly and have to be performed under standardized laboratory conditions (23). Results obtained in these tests may differ substantially from real-life conditions, as pre- and post-study glucose excursions and participant behavior are usually not documented.

Contemporary CGM devices are easily applied and worn by individuals over multiple days. The sensor is placed under the skin and measures subcutaneous glucose in contrast to venous or capillary glucose measured by patient devices, bedside devices or in the laboratory (25). The CGM devices have become widely available in recent years and the subcutaneous measurements have been shown to correlate well with both blood glucose in patients with diabetes (type 1 and 2) (26, 27), as well as in healthy individuals (7). They are easy to use and measure glucose in much shorter intervals than would be feasible to do with other devices. The precision of these devices differs from model to model (25), but is in general similar to that of point-of-care glucometers (28).

The main aim of this pilot study was to evaluate and confirm the effect of FD on glucose homeostasis in a novel real-life study design and with repeated intakes. An additional aim was to test the feasibility of our study design to be applied in future real-life trials defining the effect of different food products on glucose homeostasis in defined populations.

## Method

### Study design

The pilot study was performed at Aventure AB, Lund, Sweden. Participants attended the study facilities for one screening visit and one visit to sign informed consent, pick up food and the CGM device (Dexcom G6, San Diego, CA, United States), and receive all further instructions. The rest of the study was performed in the participants' homes.

### Ethics

This trial was approved by the Swedish Ethical Review Authority (Drn 2021–01438) on March 31, 2021 and registered at clinicaltrials.gov as NCT04848233. The authors confirm that all ongoing and related trials for this intervention are registered. It was conducted in accordance with the ethical principles that have their origins in the Declaration of Helsinki and its subsequent amendments. Written informed consent was obtained from all participants. The investigational products were produced in Sweden.

### Participants

Participants were recruited through advertising to staff at Aventure AB and other companies in the same office building. In addition, advertisement was posted once in social media. The participant profile has been defined according to findings in data from previous studies (29). Participants were required to be 40–65 years of age, have a BMI of 25.0–29.9 kg/m^2^, HOMA-IR <2.5, and fasting blood glucose <6.1 mmol/L. In addition, the participants should have been weight stable for the last 3 months and have access to an iPhone with Bluetooth 4.0 and iOS13 or newer.

### Test products and placebo

Three drinks were included in the study. The first investigational product, FD, was a commercially produced drink in form of a lightly carbonated water with a proprietary blend “molar ratios presented in (30)” of 2.6 g free amino acids (L-Leucine, L-Threonine, L-Lysine Monohydrochloride, L-Isoleucine and L-Valine, 5AA) as well as 250 mcg chromium picolinate (CrPic) (Good Idea^®^, Double Good AB, Lund, Sweden). Besides water and the active ingredients, the test product also contained natural lingonberry flavor. The placebo product was identical to the test product with respect to all ingredients, but 5AA and CrPic. A second investigational product, FDJ, was also included and besides its identical content of 5AA and CrPic, it also contained fruit juice concentrates from black currant, cranberry and apple. Both investigational products and placebo were filled in identically appearing PET-bottles and two of them (FD and placebo) matched regarding taste, texture and by being colorless. The FDJ drinks were packaged in identical bottles but had a dark red color and fruity flavor.

### Screening process

Eligibility of interested participants with respect to age, BMI and medications was reviewed at the initial contact over phone or e-mail. Those matching the criteria were invited to a screening visit. After an overnight fast of 12 h, the participants reported to the study facility in the morning of the screening visit. A nurse went through the inclusion criteria and collected information about medication, allergies, potential weight changes and family history of diabetes. Next, the height, waist circumference, weight and body composition (Tanita BC-418MA) were measured, and a capillary blood sample was taken to check fasting blood glucose (HemoCue Glucose 201 system, HemoCue, Ängelholm, Sweden). If all inclusion criteria were met, a venous blood sample was drawn from the antecubital vein for analysis (ECLIA) of serum insulin (Laboratoriekemi, Malmö University hospital, Malmö, Sweden). HOMA-IR of each participant was calculated and those still meeting the inclusion criteria were included in the study.

### Intervention

The duration of the intervention was 10 days, corresponding with the lifespan of the CGM sensor. The participants applied the sensor themselves in the morning of day 0 and had no special instructions for the rest of that day, other than making sure their glucose data was registered in the app (Nutrisense.io, IL, United States). The meal and drink schedule for all study days is shown in Table 1. The participants were asked to not eat or drink anything within 120 min of each main meal and to remain fasting from 9 pm every evening. Participants were instructed to record exact mealtimes as well as any additional snacks and drinks. Furthermore, they were instructed to keep their level of exercise and amount of sleep constant. No alcohol intake was allowed during the study. Nutrient content of meals and drinks are presented in Table 2.

**Table 1:** Meal and drink schedule.

	**day 0**	**day 1**	**day 2**	**day 3**	**day 4**	**day 5**	**day 6**	**day 7**	**day 8**	**day 9**
**Drink**	**run-in**	**Placebo**	**FD**	**FDJ**	**placebo**	**FD**	**FDJ**	**placebo**	**FD**	**FDJ**
**Breakfast**	**ad libitum**	**B1**	**B1**	**B1**	**B2**	**B2**	**B2**	**B3**	**B3**	**B3**
**Lunch**	**ad libitum**	**L1**	**L1**	**L1**	**L2**	**L2**	**L2**	**L3**	**L3**	**L3**
**Dinner**	**ad libitum**	**D1**	**D1**	**D1**	**D2**	**D2**	**D2**	**D3**	**D3**	**D3**

FD, functional drink; FDJ, FD + fruit juice.

**Table 2:** Meal composition.

**Meal/Drink**	**Description**	**Energy**	**Carbohydrates**	**Protein**	**Fat**	**Fiber**	**FDJ as % of total**
		**(kcal)**	**(g)**	**(g)**	**(g)**	**(g)**	**carbohydrates (%)**
B1	White wheat bread, butter, ham, cucumber	490.4	60.6	18.6	17.3	3.8	17.0
L1	Chicken Red Curry, rye crisp bread, butter, carrot	561.5	58.6	27.2	20.7	9.1	17.5
D1	Spicy Cajun Chicken, wheat crisp bread, butter, broccoli	487.7	45.8	27.9	19.8	11.8	21.3
B2	Thin pancakes, greek style soygurt, strawberry jam, rye crisp bread, butter, orange	517	62.1	14	23.9	2.1	16.6
L2	Meat balls, mashed potatoes, rye crisp bread, wheat crisp bread, sweet corn	787.2	53.6	26.5	48.3	9.2	18.8
D2	Panang Curry Chicken, white rice, red pepper	548.5	76.5	21.7	16.2	4.1	13.9
B3	Oatmeal, oat milk, apple, wheat crisp bread, butter	469.9	64.0	10.1	10.7	10.3	16.2
L3	Fried fish, chips, sauce, carrot	824.2	72.6	18.2	51.2	10.2	14.6
D3	Chili con Carne, white wheat bread, butter, tomato	553.2	57.2	27.8	21.4	11.8	17.8
FD	Carbonated water, 5AA and CrPic, lingonberry aroma	13.2	0.0	0.0	0.0	0.0	
FDJ	Carbonated fruit juice, 5AA and CrPic	51.9	12.4	0.3	0.1	0.8
Placebo	Carbonated water, lingon aroma	0	0	0	0	0	

B, breakfast; L, lunch; D, Dinner; FD, functional drink; FDJ, FD + fruit juice.

### Outcome measures

The pre-defined primary outcome variable, the 120-min postprandial glucose response, is best characterized by the iAUC_0−120*min*_ for subcutaneous glucose. The iAUC is calculated as the area under the curve, above the baseline level at the beginning of the meal (31). In addition, the maximum glucose value and the time to maximum glucose value within 120 min (C-max, T-max) were analyzed.

### Sample size calculation

In the previously performed meal study (24), significant effects were found in the per protocol population which consisted of 48 participants for FD and placebo, respectively, thus 96 meals in total. We estimated that to achieve a similar or higher number of meals per test drink with one run-in day and 9 study days, studying three meals a day, would require 6 participants. This setup had the potential of delivering CGM data from a total of 162 meals (54 with each drink).

### Randomization

A full-factorial design was used where all participants received all combinations of drink and meal (see Table 1). The treatments were not randomized and the meals as well as the test drinks were served in the same order for all participants.

### Statistical analysis

The trapezoid rule, a numerical integration method used to approximate the area under a curve, was used for calculating iAUC values, which is widely used to calculate the area under pharmacokinetic curves (32, 33). In cases where expected measurements were missing, simple imputation (mean of adjacent measurements) was used to substitute for these values. The differences between the drinks were obtained from the linear contrast statements of an analysis of covariance (ANCOVA), whereby only logically plausible covariates and random factors, with a significant influence (*p* < 0.05) and homogenous regression slopes (for covariates) were included in the model (34). Factors evaluated as covariates or random factors for potential inclusion in the ANCOVA were: subject and self-reported physical activity within 120 min before or after the beginning of a meal, recorded activities during the day (total daily steps, total daily running in km, total daily stair climb, total estimated daily calories burned during additional activities) as well as meal and drink characteristics (total calories, total carbohydrates, total protein, total fats, total fiber).

## Results

The participants were recruited in April 2021 and the study was completed by June 2021. With the aim to recruit 6 men and women in equivalent numbers and according to pre-defined inclusion and exclusion criteria, a total of 7 candidates were screened. One of them did not meet the cut-off for HOMA-IR and was excluded. Baseline characteristics (± SD) of the eligible participants (3 men and 3 women) were: age 49.0 (4.05) years, weight 85.7 (8.21) kg, BMI 27.1 (1.17) kg/m^2^, fasting glucose 4.82 (0.33) mmol/L, and HOMA-IR 1.77 (0.17). Half of the participants had a family history of diabetes with either one or more first or second-degree relative having diabetes type 1 or 2 (see Table 3).

**Table 3:** Participant description.

**Sex**	**Age (years)**	**Weight (kg)**	**Height (m)**	**BMI (kg/m^2^)**	**Waist (cm)**	**Fasting glucose (mg/dl)**	**HOMA-IR**	**Medication**
F	53	78.6	1.70	27.2	83	76	1.8	None
F	48	82.3	1.72	28.0	101	90	2.0	None
M	46	86.9	1.75	28.5	99	85	1.4	Antihistamin when needed
F	45	74.5	1.72	25.2	85	88	1.7	None
M	55	97.8	1.94	26.0	102	96	1.9	Enalapril 5mg 1/day, Iron supplement
M	47	94.0	1.84	27.8	101	86	1.8	None

**Figure 1:**
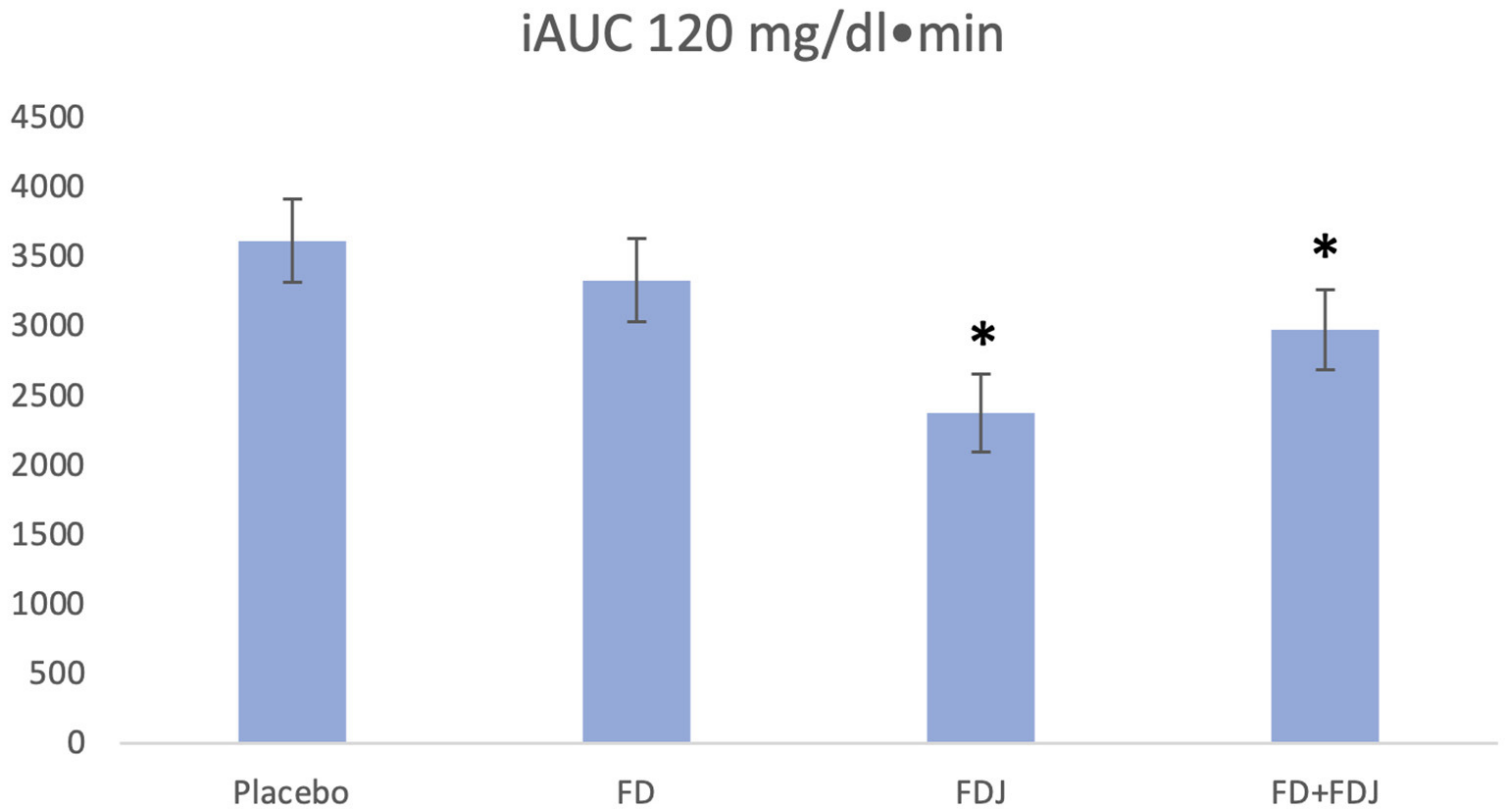
iAUC_0−120_ values adjusting for subject and physical activity in the 120 min before or after the beginning of a meal. Covariates appearing in the model are evaluated at the following values: total carbohydrates = 65.3 g, total protein = 21.3 g, total fats = 25.5 g. *p < 0.001.

A total of 14.894 measurements was obtained from the CGM devices and 156 meals were available for analysis. The ANCOVA analysis showed a significant 34% reduction of iAUC_0−120*min*_ for FDJ (*p* < 0.001), with iAUC_0−120*min*_ for placebo being 3.612 mg/dl · min (95% CI: 3.022 mg/dl · min−4.203 mg/dl · min) and 2.378 mg/dl · min (95% CI: 1.820 mg/dl · min−2.935 mg/dl · min) for FDJ. FD on its own did not show a significant reduction of iAUC_0−120*min*_ (3.351 mg/dl · min; 95% CI: 2.742 mg/dl · min−3.919 mg/dl · min) (see Figure 1). Covariates included in the model and used to derive the results above were carbohydrates at 65.3 g, protein at 21.3 g and fats at 25.5 g; random factors included in the model were subject number as well as self-reported physical activity within 120 min before or after the beginning of a meal. When pooling the data of FD and FDJ in the same model, a significant reduction of 17.6% was documented for FD/FDJ (*p* = 0.007).

C-max of postprandial glucose within 120 min was 153.8 mg/dl (95% CI: 147.6 mg/dl−160.0 mg/dl) for placebo, 145.4 mg/dl (95% CI: 138.9 mg/dl−151.7 mg/dl) for FD and 132.8 mg/dl (95% CI: 125.7 mg/dl−139.8 mg/dl) for FDJ. The 14% difference in C-max between placebo and FDJ was significant (*p* = 0.001), whereas the 6% difference between placebo and FD was borderline significant (*p* = 0.053). The ANCOVA model for assessing C-max differed slightly to the model for iAUC_0−120*min*_, it did not include total protein and physical activity before and after the meal as covariates, since these were not significant. The included covariates were assessed at following levels: total carbohydrates at 65.3 g and total fats at 25.5 g. When pooling FD and FDJ data and comparing them to placebo, the ANCOVA model remained unchanged with regards to included variables and the C-max difference between drinks was 9% and significant (FD/FDJ: 139.6 mg/dl vs. placebo 152.8 mg/dl, *p* = 0.001). No significant differences were found for T-Max. No significant differences were found for the change from baseline to peak subcutaneous glucose. Participants were within the physiological target range as defined by the American Diabetes Association for subcutaneous glucose measurements (35, 36) in 98% of the time.

## Discussion

With FD and FDJ both containing the same active ingredients and mainly differing in the carbohydrate content, the results of this study confirm the postprandial glucose lowering efficacy of the amino acids and chromium combination, using CGM devices. While no significant reduction in postprandial glycemia was found when comparing the FD to placebo on its own, FD with added carbohydrate-containing juice (FDJ) as well as the combination of FD and FDJ, demonstrated a pronounced and significant effect on iAUC_0−120_ (adjusted for the meal composition). Higher efficacy of FDJ compared to FD suggests an enhanced postprandial glucose lowering with an increasing carbohydrate load, a situation in which the improved early insulin response previously shown for FD (without changing total iAUC for insulin) (24, 29) could be even more beneficial for postprandial glucose disposal. The positive effects of early insulin have convincingly been shown both in healthy individuals and diabetes patients (37, 38). In addition, effects of FD on GLP-1 and GIP could influence the effects observed in this study (24, 39). The current results also show that the effect of FD/FDJ on postprandial glucose is sustained and can be achieved in repeated meals over the course of the day.

The study design proved successful and efficient. The participants themselves were to a large extent responsible for the data collection and the adherence to the protocol was good. The time from ethical approval to finalized data collection was substantially shorter (appr. 2 months) than the normal time needed for a meal study (4–6 months). With only 6 individuals, a large dataset for the equivalent of 156 standard laboratory-based meal test was collected. For the iAUCs to be comparable, adjustments for the meal composition as well as other factors were necessary and ANCOVA appeared to be the appropriate model. The factors affecting iAUC values were all expected and physiologically plausible. The variable for physical activity within 120 min of the beginning of a meal was relatively rough, i.e., “did a physical activity take place or not”, yet still played an important role and was thus included in the model as a random factor. Activity within 120 min before the meal reduced the pre-meal glucose value and thus increased the increment, whereas activity within 120 min after beginning of the meal reduced the increment. Overall, daily activity levels did not show significant differences in iAUCs of the individual meals, at least in this small group of subjects. It therefore seems necessary for future studies to improve the monitoring of physical activity, for example by adding data on heart rate from wearables and include it as an additional covariate. A major limitation of this study is the use of two FD with a different nutrient content while using only one comparator matched for FD (and not FDJ). It was not the primary aim of this study to define effects of FDJ on postprandial glucose but rather to evaluate the novel methodological approach. The albeit significant effects shown for FDJ in analyses adjusted for carbohydrate content of the juice need to be studied in follow-ups including an adequate comparator utilizing the novel CGM-based approach presented.

Since even this small pilot trial in 6 participants and repeated meals with a varying macronutrient content delivered significant results for postprandial glucose acquired by CGM, it may be possible to perform such studies in even closer to real-life settings. Subjects could eat meals of their choice, as long as the nutrient content is known or can be estimated with a high precision. The differences between FD and comparator missed statistical significance, which was shown in a previous controlled trial (24). This implies that less standardized meals will likely require larger datasets for postprandial glucose than studied herein, which can easily be achieved in the presented study design. Increasing the number of individuals studied, the use of CGM as well as mobile applications for food and tracking physiological variables would also enable comparisons of inter-individual differences in glucose responses and generate more robust data that is highly desirable for the study of true effects of foods on glucose metabolism.

## Conclusions

Taken together, the data presented in this pilot study indicate that continuous subcutaneous glucose measurement delivers a quantity of quality data, which is sufficient to detect effects of a functional drink previously shown to lower postprandial glucose in apparently healthy, overweight subjects. The results imply, that it could be possible to perform studies even closer to real-life by further relaxing meal standardization alongside an improvement in the quality of data on physical activity, as long as precise data on meal composition is collected.

## Data availability statement

The raw data supporting the conclusions of this article will be made available by the authors, without undue reservation.

## Ethics statement

The studies involving human participants were reviewed and approved by Swedish Ethical Review Authority. The patients/participants provided their written informed consent to participate in this study.

## Author contributions

AS, PH, and EÖ all took part in the conceptualization and design of the study. The practical work and data collection was performed by EÖ and the statistical analysis was done by AS. All authors contributed to the interpretation of data and writing of the manuscript.

## Funding

The study was funded by Double Good AB.

## Conflict of interest

This study received funding from Double Good AB. The funder had the following involvement with the study: EÖ is co-inventor of the patent family describing the functional drink studied, and works for Aventure AB/Double Good AB, that has a license to use the patent. starScience GmbH AS and PH have received funding for other studies by Aventure AB/Double Good AB. PH holds shares of Double Good AB. AS is employed by starScience GmbH.

## Publisher's note

All claims expressed in this article are solely those of the authors and do not necessarily represent those of their affiliated organizations, or those of the publisher, the editors and the reviewers. Any product that may be evaluated in this article, or claim that may be made by its manufacturer, is not guaranteed or endorsed by the publisher.
